# Obituary

**Published:** 1869-12

**Authors:** 


### OBITUARY.

James Gamble, D. D. S., died of consumption on the 17th of November, 1869, at the residence of Mr. Thomas Milligan, in Fayette county, Ind. He was born in Preble county, Ohio, in 1836; soon afterwards his parents removed to Monroe county, Ind., which latter place he continued to make his home.

Dr. Gamble received his diploma from the Ohio College of Dental Surgery in the Spring of 1869, and, although doubly afflicted, he has during his brief Professional life, proven himself worthy of the honors conferred upon him by that institution. C.

